# Probing interspecies metabolic interactions within a synthetic binary microbiome using genome-scale modeling

**DOI:** 10.20517/mrr.2023.70

**Published:** 2024-05-27

**Authors:** Kiumars Badr, Q. Peter He, Jin Wang

**Affiliations:** Department of Chemical Engineering, Auburn University, Auburn, AL 36849, USA.

**Keywords:** Synthetic microbiome, methanotroph-photoautotroph coculture, interspecies metabolic interactions, genome-scale metabolic modeling, steady state modeling, dynamic modeling

## Abstract

**Aim:** Metabolic interactions within a microbial community play a key role in determining the structure, function, and composition of the community. However, due to the complexity and intractability of natural microbiomes, limited knowledge is available on interspecies interactions within a community. In this work, using a binary synthetic microbiome, a methanotroph-photoautotroph (M-P) coculture, as the model system, we examined different genome-scale metabolic modeling (GEM) approaches to gain a better understanding of the metabolic interactions within the coculture, how they contribute to the enhanced growth observed in the coculture, and how they evolve over time.

**Methods:** Using batch growth data of the model M-P coculture, we compared three GEM approaches for microbial communities. Two of the methods are existing approaches: SteadyCom, a steady state GEM, and dynamic flux balance analysis (DFBA) Lab, a dynamic GEM. We also proposed an improved dynamic GEM approach, DynamiCom, for the M-P coculture.

**Results:** SteadyCom can predict the metabolic interactions within the coculture but not their dynamic evolutions; DFBA Lab can predict the dynamics of the coculture but cannot identify interspecies interactions. DynamiCom was able to identify the cross-fed metabolite within the coculture, as well as predict the evolution of the interspecies interactions over time.

**Conclusion:** A new dynamic GEM approach, DynamiCom, was developed for a model M-P coculture. Constrained by the predictions from a validated kinetic model, DynamiCom consistently predicted the top metabolites being exchanged in the M-P coculture, as well as the establishment of the mutualistic N-exchange between the methanotroph and cyanobacteria. The interspecies interactions and their dynamic evolution predicted by DynamiCom are supported by ample evidence in the literature on methanotroph, cyanobacteria, and other cyanobacteria-heterotroph cocultures.

## INTRODUCTION

In nature, almost all microorganisms exist in complex microbial communities, where interactions among different members stabilize the structure and functionalities of the communities under various environmental stresses^[1]^. Thanks to a higher degree of freedom and a larger pool of genes, microbial communities offer many advantages, including efficient utilization of substrates and increased productivity through division of labor, as well as enhanced robustness against perturbations^[2-7]^. Natural microbial communities have long been utilized by humans in the traditional food fermentation processes, arising independently in multiple ancient cultures as far back as 7,000 BC^[8,9]^. Recently, natural microbial communities have also been widely used in wastewater treatment processes and bioremediation. However, most currently established biotechnologies utilize axenic cultures to produce bulk chemicals and other valuable products, such as organic acids, antibodies, and pharmaceuticals^[10,11]^. This is mainly due to the simplicity associated with the modeling, monitoring, and control of axenic cultures. In the past decade, synthetic microbial communities have drawn increasing research interests and seen more applications^[12]^. Below, we provide a brief review of recent advancements in this area.

### Recent applications of synthetic microbiomes in biotechnology and bioprocessing

The advantages offered by microbial communities have drawn increasing research interest in using synthetic microbial communities for different applications. One of them is the production of novel chemicals that cannot be produced by monocultures, such as chemicals that exhibit anti-microbial activities and can only be found in microbial communities^[12-16]^. Another example is the consolidated bioprocessing of lignocellulose, where consortia consisting of a cellulose-degrading strain and a chemical-producing strain have demonstrated superior performance over their monocultures^[17,18]^. Another important application of synthetic microbial consortia is the conversion and valorization of biogas (containing 50%~70% CH_4_, 30%~40% CO_2_, and trace amounts of other gases such as H_2_S and NH_3_). In nature, microbial communities provide highly efficient energy recovery and carbon recycling from naturally produced biogas. This is achieved through the metabolic coupling of methane oxidation to oxygenic photosynthesis^[19-21]^. Inspired by how natural microbial communities recycle carbon and recover energy, many synthetic methanotroph-photoautotroph (M-P) cocultures have been explored for biogas conversion^[22-28]^. The increasing application of synthetic microbiomes further drives the fundamental understanding of the interspecies interactions, which lays the foundation for further engineering of these synthetic microbiomes to improve performance and optimize operation conditions.

### State of the art on the understanding of interspecies interactions within microbiomes

It has been known that the metabolic interactions among different species in a microbial community play a significant role in determining the structure, composition, and function of the microbiome^[29-31]^. Different interactions, through unidirectional or bidirectional exchange of metabolites or charged compounds, result in different symbiotic relationships, including mutualism, amensalism, commensalism, neutralism, and parasitism^[32]^. Interspecies interactions and symbiotic relationships within a community are also known to exhibit dynamic shifts under different environmental conditions, which contribute to the resilience and robustness of the microbial community^[33,34]^. Recent advances in meta-omics (metagenomics, metatranscriptomics, metaproteomics and metabolomics) have produced a plethora of data on the composition and activity of microbial communities in different environments^[35]^. However, these meta-omics datasets are usually highly complex and contain system-wide responses/variations, which makes the integration and interpretation of them very challenging^[36-41]^. Specifically, despite the recent advances, little is known about how different factors would determine the interactions within a community, not to mention how these interactions evolve in response to environmental and genetic perturbations. On the other hand, synthetic communities, especially binary communities that consist of two well-defined species, could serve as useful model systems to understand microbial interactions. As the strains in a synthetic community do not necessarily have a chance for co-evolution, binary synthetic communities provide an opportunity to understand how emergent mutualistic interactions establish and evolve over time. In this work, we use a binary M-P coculture as the model system to understand the establishment of emergent mutualistic interactions and the evolution of interspecies interactions over time. To help address the challenges with experimental studies of microbiomes, this work explores an *in-silico* approach to probe the interactions within the model M-P coculture.

### Genome-scale metabolic models

Genome-scale metabolic models (GEMs) have been recognized as a valuable and effective tool to help elucidate cellular metabolisms. They provide a foundation to integrate various (meta)-omics data and gain novel insight into the structure and functionality of the microbiomes^[42-45]^. A GEM is an organism-specific comprehensive knowledge base of cellular metabolisms, which consists of an organized list of metabolic reactions reconstructed from an annotated genome. In essence, a GEM is the stoichiometric matrix of all potential reactions within a cellular metabolic network, together with a set of physiochemical and condition-specific constraints on the reaction fluxes^[42,46]^. Among different modeling approaches, flux balance analysis (FBA) is the most commonly applied constraint-based approach to predict the flux distribution of the organism under a given growth condition. As the number of reactions is usually (much) larger than the number of metabolites in a GEM, FBA relies on (linear) optimization to select a flux distribution among an infinite number of feasible solutions, usually by maximizing biomass growth^[46]^.

In the last decade, steady-state and dynamic GEMs have been proposed to model microbial communities^[39]^. The steady-state modeling approaches, such as SteadyCom^[47]^, usually take a compartmentalized approach, where each species is modeled as a compartment in the overall system, and a community compartment is available for the exchange of metabolites among members^[47-50]^. On the other hand, the dynamic modeling approaches, such as dynamic flux balance analysis (DFBA), also assume the cellular metabolism is always in a quasi-steady state whose evolvement is driven by the dynamics of the environment (e.g., bioreactor)^[51-54]^. In this way, both the steady-state and the dynamic GEM approaches eliminate the need for kinetic parameters of the intracellular reactions. In recent years, there has been a significant increase in the application of community GEMs of different sizes^[39]^. However, both modeling approaches have their limitations. The steady-state GEMs can predict the interspecies interactions but cannot capture their dynamic evolution, while the dynamic GEMs can capture the overall system dynamics but cannot predict the interspecies interactions.

**Figure 1:**
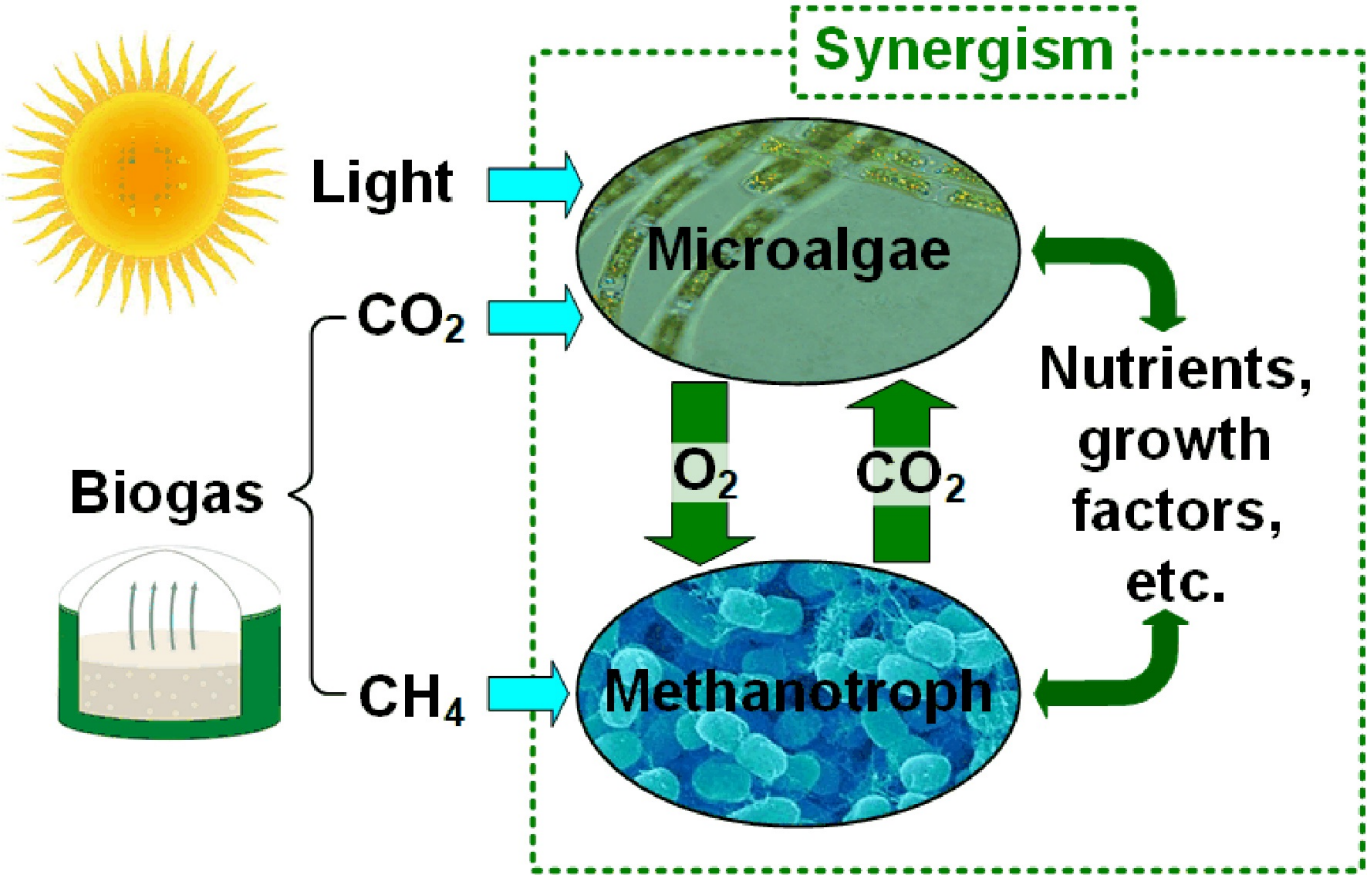
Schematic of known and speculated interactions in a methanotroph-microalgae coculture.

In this work, using a methanotroph-cyanobacteria coculture pair as the model system, we present a dynamic GEM approach, namely DynamiCom, that can predict the evolution of the emergent interspecies interactions within the binary microbiome. In our previous work, we assembled and investigated several different M-P cocultures that exhibit stable growth under varying substrate delivery and illumination regimes^[22,23,55]^. The interspecies interactions within M-P cocultures using biogas as the substrate include the known cooperative interactions (or mutualism) between the two partners, i.e., the exchange of in situ produced O_2_ and CO_2_ and additional unknown interactions as illustrated in Figure 1. In our previous work, we have demonstrated that for the coculture of *Methylomicrobium buryatense* (*M. buryatense*) 5GB1 - *Arthrospira platensis* (*A. platensis*), the enhanced growth observed for both species in the coculture cannot be fully explained by the in-situ exchange of O_2_ and CO_2_, confirming the existence of other unknown metabolic interactions for the coculture^[22]^. In this work, using *M. buryatense* 5GB1 - *A. platensis* as the model system, we examine different modeling approaches to identify and understand the emergent synergistic interspecies interactions.

## METHODS

In this section, we introduce the GEMs used in this work and briefly discuss their implementations. All simulations in this work were conducted using Matlab (ver. R2021a), with COBRA Toolbox and linear solver “glpk”. All GEMs and simulation codes can be found in the Github repository (links can be found in the Declarations section).

### GEMs of *M. buryatense* 5GB1 and *A. platensis*

In both the steady-state and dynamic GEM approaches for microbial communities, high-quality GEMs are needed for each community member. The GEMs used in this work are based on the published models, iMb5G(B1) for *M. buryatense*^[56]^ and NIES-39 for *A. platensis*^[57]^. Both published models were refined in this work using the system identification-based framework we previously developed^[58]^.

For iMb5G(B1), the following modifications were made: the productions of different organic acids (formic, acetic, lactic, *etc.*) were decoupled from the biomass reaction so that they can be freely excreted by the model; the fermentation reactions reported by Gilman *et al.* were added to the model^[59]^. More details about the modified iMb5G(B1) can be found in our previous publication^[60]^.

For NIES-39, many modifications were made in this work to reflect recent findings in the literature^[61,62]^, including photosynthesis and electron transport chain, Calvin/cycle/Pentose phosphate pathways, and Pyrimidine/serine/ glutamate metabolism. Supplementary Table 1 provides a list of major modifications to the model.

### Steady-state community GEM - SteadyCom

In this work, we chose SteadyCom as the steady-state GEM approach for the M-P coculture. The implementation for SteadyCom can be found at https://github.com/maranasgroup/SteadyCom.

After combining the modified iMb5G(B1) and NIES-39, there are more than 1,300 reactions in the coculture model. To simplify the analysis without affecting the model prediction, we first identified dead-end reactions that cannot carry any fluxes under any conditions and removed them. The reduced coculture model contains 579 reactions, less than half of that in the original model. In order to develop a coculture model where metabolites can freely exchange, the metabolite names from both models have to be the same. Therefore, the metabolite names of both models were modified to follow the same naming convention. To model the potential interactions between the two strains, we added a community compartment ([u]), and the metabolites that were defined in the extracellular compartment ([e]) have to be able to move freely in and out of the community compartment. This was achieved by adding transport reactions between [e] and [u]. For example, for acetate in the extracellular compartment of methanotroph, denoted as M1ac[e], the following transport reaction was added: M1ac[e] 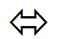 ac[u].

**Figure 2:**
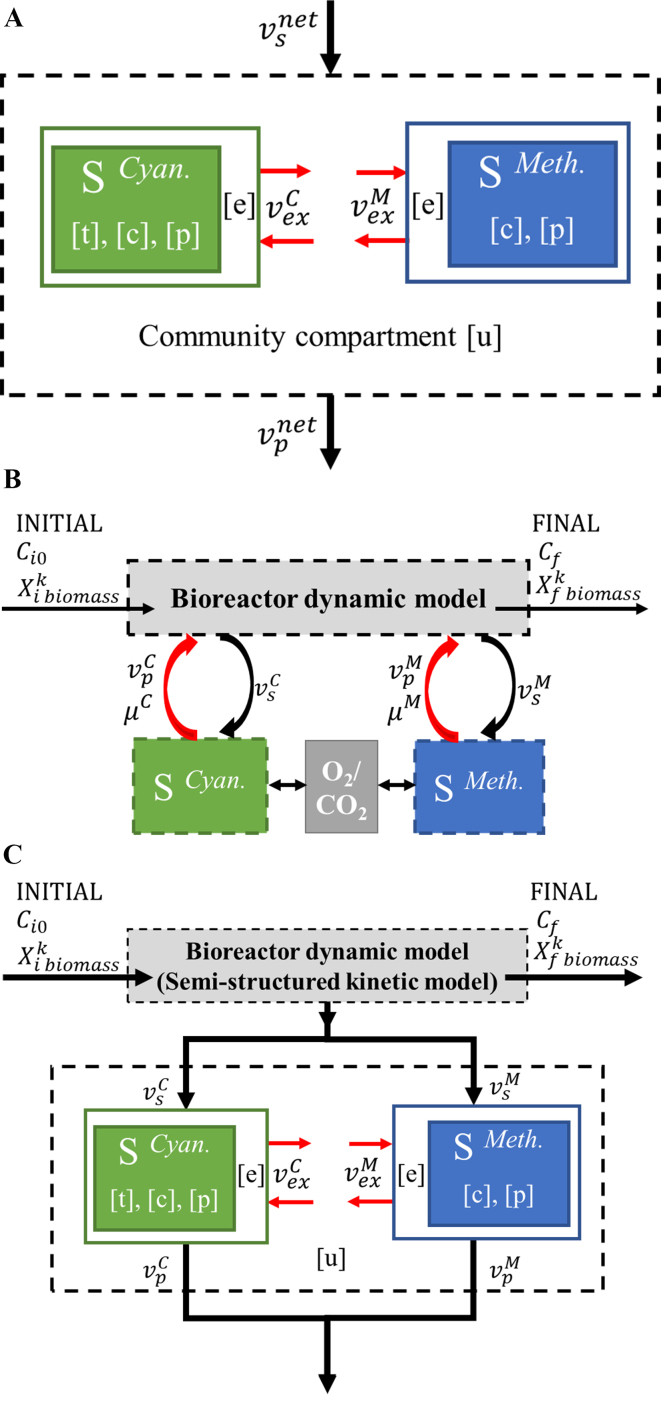
Modeling schematics for the M-P coculture. (A) SteadyCom; (B) DFBA Lab; (C) the proposed DynamiCom. M-P: Methanotroph-photoautotroph; DFBA: dynamic flux balance analysis.

A schematic setup of the SteadyCom for the model M-P coculture is shown in Figure 2A, where the two colored boxes represent the intracellular environment for the cyanobacteria (green) and methanotrophs (blue), respectively. Compartment [u] is the shared community compartment, which supplies the nutrients and houses the metabolites excreted by each organism. The community compartment allows the exchange of metabolites and the uptake of nutrients by both species in the community. The objective function for SteadyCom is usually maximizing the community biomass production, which is a (weighted) summation of individual organisms in the microbiome. 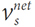 and 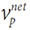 are the net substrate consumption and net product excretion by the community; 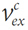 and 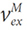 are the exchange fluxes of the cyanobacteria and methanotrophs, respectively.

### Dynamic community GEM - DFBA Lab

In this work, we use DFBA Lab as the dynamic GEM approach for the M-P coculture. The implementation can be found at http://yoric.mit.edu/dfbalab. DFBA Lab is an advanced implementation of dynamic FBA that addresses a key challenge with FBA - non-unique solutions. Because the number of reaction fluxes (i.e., the unknowns) is much larger than the number of constraints, there are often non-unique solutions to the optimization problem. It is quite often that the *in silico* optimal solution may switch among different non-unique solutions that all optimize the objective function, e.g., maximizing biomass growth. Even though each optimal solution (i.e., flux distribution) is a feasible solution to the FBA, switching among different optimal flux distributions between two consecutive time points is not possible *in vivo*. DFBA Lab implements lexicographic optimization to obtain unique exchange fluxes at different time points and ensures a continuous dynamic response of the cellular flux shift over time.

Figure 2B illustrates the setup of the DFBA model for the M-P coculture, where each organism is treated as an independent component, and there is no shared community compartment. The interaction between different species is captured indirectly through the dynamics of the macroscopic environment, i.e., the bioreactor. In Figure 2B, *μ^C^* and *μ^M^* are the growth rates, 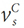 and 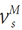 are the substrate uptake rates, and 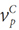 and 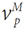 are the product excretion rates for the cyanobacteria and methanotrophs, respectively. In DFBA, for each time instant, the substrate uptake rates are computed based on an empirical model, usually Michaelis-Menten equation, using the substrate concentration in the bioreactor; then FBA is applied to predict the intracellular flux distribution using substrate uptake rates as additional constraints; finally, the FBA predicted cell growth rates and product excretion rates, as well as the substrate consumption rates, are fed to the dynamic model for the bioreactor, which is solved to update the substrate, product and biomass concentrations for the next time point.

It is worth noting that in DFBA, there is no shared compartment for the community members to exchange metabolites; therefore, DFBA cannot predict emergent interspecies interactions. In this work, to capture the synergistic effect caused by the known cross-feeding of CO_2_ and O_2_ within the M-P coculture, we manually added the exchange of these molecules.

### Semi-structured kinetic modeling

In DFBA, simple substrate uptake kinetics, i.e., the Michaelis-Menten equation, dictate the overall dynamics of the coculture. Such simplified treatment does not consider the potential emergent interactions between the two species in the coculture, which could result in failure to accurately predict the growth dynamics of the coculture. For the M-P coculture, even with the manually added CO_2_/O_2_ exchange, DFBA predictions could not capture the enhanced growth of both species in the coculture, as shown in the Results section. To better capture the growth dynamics of the M-P coculture, we recently developed a semi-structured kinetic model, which includes the biomass growth of photoautotrophs and methanotrophs, as well as the mass balance in the gas and liquid phases. The growth of both species depends on the substrate concentrations in the liquid phase, which is linked to the gas phase concentrations through mass transfer between, and mass balance within, the gas and liquid phases. Various experiments have shown that the semi-structured kinetic model can accurately predict coculture growth under a wide range of growth conditions^[55]^.

In general, kinetic models that use Monod-like equations to describe microbial growth are considered unstructured, as no intracellular details are considered in the model. In our kinetic model for the M-P coculture, the cross-feeding of O_2_ and CO_2_ between the methanotroph and cyanobacteria was explicitly considered, which is why the model is termed “semi-structured”. As the semi-structured kinetic model can accurately predict the coculture growth, we will use the substrate uptake rates and product excretion rates predicted by the model as additional constraints for FBA to predict intracellular flux distributions.

### The improved dynamic community GEM - DynamiCom

To address the limitation associated with DFBA, i.e., no community compartment to enable interspecies interactions, we propose an improved dynamic community GEM, termed DynamiCom. The model setup for the M-P coculture is shown in Figure 2C. The basic structure of the DynamiCom is similar to DFBA, where the intracellular details are captured by a steady-state GEM of the coculture, while the system dynamics is determined by the dynamics of the bioreactor. However, there are some key differences between DynamiCom and DFBA, which allows DynamiCom to predict the evolution of the interspecies metabolic interactions. First, in DynamiCom, SteadyCom is applied to compute the intracellular details of the coculture. The shared community compartment enables the prediction of the interspecies interactions within the coculture under a given condition. Second, the semi-structured kinetic model for the M-P coculture is deployed to compute the substrate uptake rates, product excretion rates, and growth rates for each organism in the coculture. Third, and more importantly, the communication between the reactor dynamics and steady-state GEM is unidirectional, indicated by the black arrows from the dynamic model to the SteadyCom. In other words, there is no feedback from the SteadyCom predictions (i.e., individual growth rates and product excretion rates) back to the dynamic model. Instead, the bioreactor dynamics is fully determined by the semi-structured kinetic model, while the substrate update rates and product excretion rates for each species predicted by the semi-structured kinetic model are fed to SteadyCom as additional constraints.

The implementation of DynamiCom is straightforward by integrating SteadyCom with the semi-structured kinetic model. At every sampling/time point, the outputs from the kinetic model (i.e., substrate uptake rates and product excretion rates for each organism) are fed to SteadyCom as the additional constraints. As the GEM of each species does not include any regulatory mechanisms, adding additional constraints will reduce the feasible space of FBA and could improve the model predictions on the interspecies interactions. Therefore, we expect that the evolving constraints predicted by the semi-structured kinetic model, which has been experimentally validated, could lead to more reliable prediction of the dynamic evolution of interspecies metabolic interactions.

## RESULTS

In this work, using the batch growth data reported in our previous work for *M. buryatense* - *A. platensis* coculture^[22]^, we compare the three GEM approaches, i.e., SteadyCom, DFBA Lab, and DynamiCom, in predicting the intra- and intercellular metabolic details for the coculture.

### Validation of the refined iMb 5G(B1) and NIES-39

The sequential single culture data reported previously (figure 6 of our previous work^[22]^) were used to validate the refined GEMs for both strains. Following the literature^[22,61]^, the non-growth-associated maintenance energies were set to 10.6 and 0.6 mmol ATP/(gDCW∙hr) for iMb 5G(B1) and NIES-39, respectively, while the growth-associated maintenance energies were set to 23 and 40 mmol ATP/(gDCW∙hr), respectively.

FBA was applied to simulate single culture growth with maximizing biomass production as the objective function. Because the defined medium was used in the experiment, the models were allowed to uptake the nutrients provided in the defined media freely, including Fe^2+^, Cu^2+^, Mg^2+^, Pi, NO_3_^-^, SO_4_^2-^ for both models and, additionally, Vitamin B_12_ for NISE-39. For the methanotroph, the experimentally measured CH_4_ and O_2_ uptake rates were utilized as the constraints; the model-predicted cell growth rate was compared with experimental measurements to validate the model’s accuracy. For cyanobacteria, the experimentally measured CO_2_ uptake rate was used as a constraint, while the constraint on the photon uptake rate was determined by performing *in silico* experiments and comparing it with the experimental growth rate for different CO_2_ uptake rates at the given light intensity. The model-predicted O_2_ production and cell growth rates were compared with the experimental data to validate the GEM accuracy. Table 1 lists the simulation setup and comparison results, showing that the model predictions agree with the experimental measurements very well. The unit for different fluxes is mmol/(gDCW∙hr), and the unit for cell growth rate is hr^-1^.

**Table 1:** Simulation setups (i.e., constraints) and performance evaluation (i.e., predicted *vs.* measured)

**Strain**	**Constraints**	**Model prediction**	**Experimental measurement**
iMb 5G(B1)	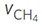 = -3.134 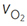 = -4.314	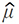 = 0.0206	*μ* = 0.0214
NIES-39	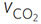 = -6.420	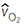 = 0.888 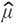 = 0.0143	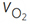 = 0.858 *μ* = 0.0152

### SteadyCom

The experimental data reported previously (figure 2 of our previous work^[22]^) were used to validate the SteadyCom model for the coculture. As SteadyCom assumes the microbial community has reached a steady state, we used the average of the measurements taken between 48-64 h to compute the inputs to the GEM, as the coculture growth rate during this segment was relatively stable. In SteadyCom, gDCW represents grams of dry cell weight for all biomass in the coculture, and the total substrate consumption rates by the community are applied as the additional constraints. In this work, the net CO_2_ consumption rate [i.e., -0.680 mmol/(gDCW∙hr)] and photon uptake rate [i.e., -6.98 mmol/(gDCW∙hr)] were used as the constraints for SteadyCom. In addition, the net O_2_ consumption rate was set to zero, as no O_2_ was detected throughout the growth experiment. In other words, the O_2_ produced by *A. platensis* must be completely consumed by *M. buryatense*. The consumption rate of CH_4_ was determined by the model based on the availability of O_2_. The other nutrients available from the defined medium were allowed to be freely uptaken by the model, similar to the case for the single cultures.

To evaluate the reliability of the SteadyCom predictions for the model coculture pair, we compare the system-level predictions by the model with experimental measurements. Table 2 summarizes the SteadyCom predicted population ratio, coculture growth rate, and CH_4_ and O_2_ consumption rates compared with their corresponding experimental measurements. It can be seen that the predictions from SteadyCom agreed very well with the experimental measurements. The accuracy of the systems-level prediction suggests that the intra- and intercellular details predicted by the model could offer insights into how the interspecies interactions affect the growth of the M-P coculture. This result also supports the use of SteadyCom in the proposed DynamiCom to predict the metabolic details.

**Table 2:** SteadyCom model predictions *vs.* experimental measurements

	**Model prediction**	**Experimental measurement**
**Population composition (%) (M:P)**	22:78	21:79
**Growth rates (hr^-1^)**	0.0190	0.0194
**CH_4_ consumption rate (mmol/gDCW/hr)**	0.664	0.665
**O_2_ consumption rate (mmol/gDCW/hr)**	0.866	0.848

**Figure 3:**
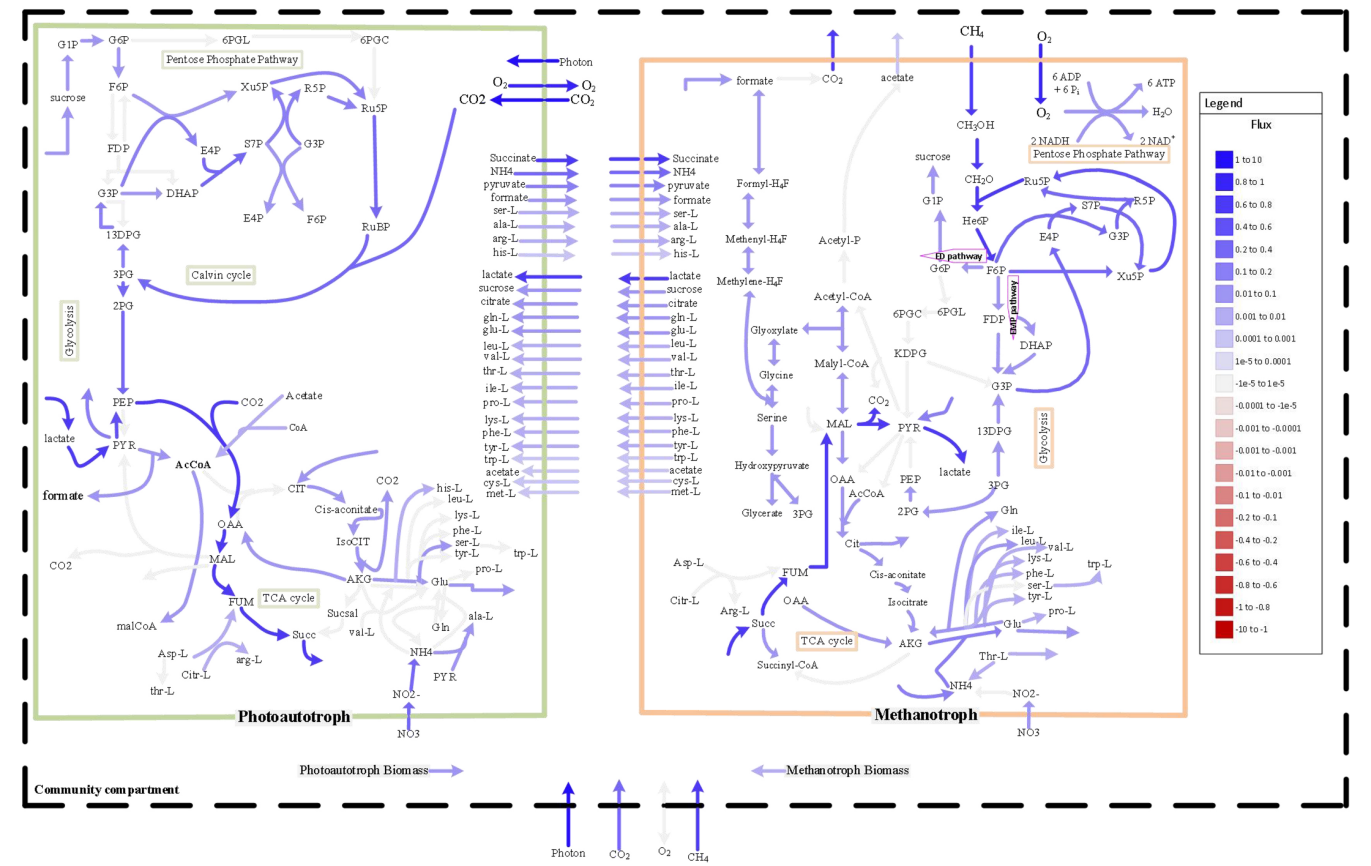
Schematic representation of the simulated metabolism of the coculture system by SteadyCom.

Besides the known cross-feeding of O_2_ and CO_2_, SteadyCom predicts a list of exchanged metabolites that contributed to the improved growth of both species in the coculture. Figure 3 depicts the metabolic cross-feeding fluxes predicted by SteadyCom within the coculture. It is worth noting that as *M. buryatense* and *A. platensis* did not have a chance to co-evolve, the metabolic interactions predicted by SteadyCom are emergent interactions that happen spontaneously. The predicted cross-fed metabolites included metabolites in the central carbon metabolic network, nitrogen sources (ammonium in particular), and a range of amino acids. It is worth noting that in SteadyCom and most community GEM approaches, the base unit for flux is the unit mass of the community, or coculture in this case, instead of individual species in the community. Therefore, the fluxes of the exchanged metabolites would have the same magnitude for the producer and the consumer. However, as the population ratio is usually not 1:1 for *M. buryatense* - *A. platensis* culture, the actual fluxes for each strain, with the strain’s unit mass as the base, would be different.

To examine the consistency of the model-predicted interspecies interactions, we have tested several different *in silico* setups for SteadyCom. Specifically, we added different constraints on which metabolites were allowed to be exchanged within the coculture. Our *in silico* experiments showed that key metabolites in the tricarboxylic acid cycle (TCA) cycle should be excluded from the exchange, otherwise no feasible solution would exist for SteadyCom. Table 3 provides the details of the allowed and excluded metabolites for exchange. Among different *in silico* setups, SteadyCom consistently predicted the same set of top metabolites (with slightly different orders in terms of cross-feeding fluxes) to be exchanged between the methanotroph and cyanobacteria. The top eight exchanged metabolites predicted by SteadyCom are: succinate, ammonium, pyruvate, formate, citrate, sucrose, glutamine, and glutamate. This result agrees with the literature on other microbial communities, where metabolites involved in the TCA cycle and amino acids were reported as dominant cross-fed metabolites^[63]^.

**Table 3:** *In silico* setups for SteadyCom

**Setup**	**Included metabolites**	**Excluded metabolites**
1	Malate/pyruvate	Succinate, oxaloacetate, fumarase, alpha-ketoglutarate
2	Pyruvate	Succinate, malate, oxaloacetate, fumarase, alpha-ketoglutarate
3	Pyruvate/succinate	Malate, oxaloacetate, fumarase, alpha-ketoglutarate
4	Succinate	Pyruvate, malate, oxaloacetate, fumarase, alpha-ketoglutarate
5	Malate/succinate	Pyruvate, oxaloacetate, fumarase, alpha-ketoglutarate
6	Malate/alpha-ketoglutarate	Succinate, oxaloacetate, fumarase, pyruvate

As shown in Figure 3, cyanobacteria provide the main favorable carbon source, such as succinate, and nitrogen source, such as ammonium, for methanotrophs. On the other hand, methanotrophs produce more amino acids for both organisms. It is likely that methanotrophs can produce amino acids at a lower cost (in biological/thermodynamical terms) than cyanobacteria, which is supported by previous work showing that methanotrophs have the advantage of producing TCA-derived products^[64,65]^.

### DFBA Lab

**Figure 4:**
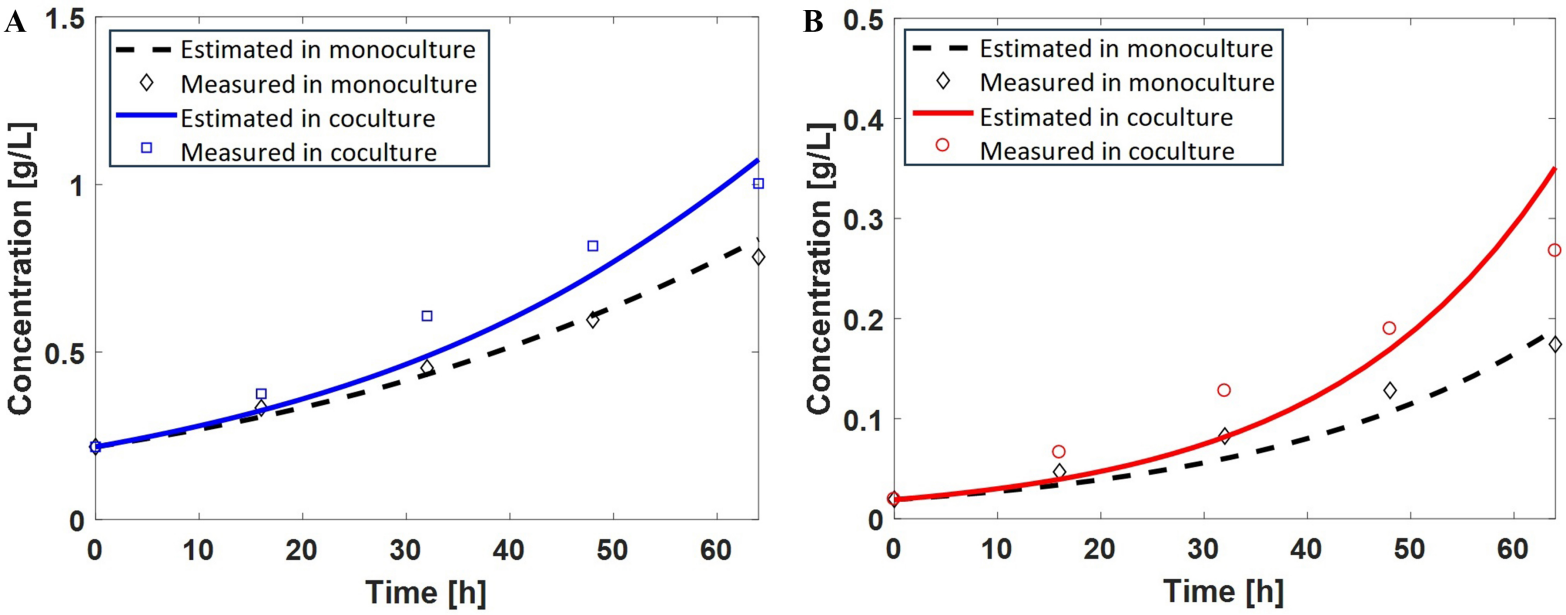
The biomass concentrations of monoculture (dashed lines) predicted by DFBA Lab agree well with experimental measurements (diamonds) for both (A) cyanobacteria and (B) methanotrophs. The biomass concentrations in coculture (solid lines) predicted by DFBA Lab have greater deviations from experimental measurements (squares and circles) for both cyanobacteria and methanotrophs, even after adjusting growth- and/or non-growth-associated maintenance energies. DFBA: Dynamic flux balance analysis.

For the dynamic GEM approach, we first tested DFBA Lab to predict the batch growth for the monocultures of *M. buryatense* and *A. platensis* using the data reported previously (figure 6 of our previous work^[22]^) for the whole experimental duration. This is to ensure that the tuning parameters in the GEM, i.e., growth and non-growth associated maintenance energy (GAM and NGAM), and substrate update kinetics were proper. As shown in Figure 4, the DFBA Lab predictions (dashed lines) for both monocultures agree well with experimental measurements (diamonds). However, when DFBA Lab was implemented for the coculture, even with manually added exchange of CO_2_ and O_2_, the model predictions (not shown) failed to track the growth of both species in the coculture. The model predictions were much lower than the measurement for both species, as the coculture model failed to capture the synergistic interactions within the coculture. To improve the model predictions, the growth- and/or non-growth-associated maintenance energies were lowered for both species, which allows for improved growth yield and better agreement with the measurements (solid lines in Figure 4). Table 4 provides the GAM and NGAM values used in the DFBA Lab models. However, model predictions still do not track the trend of the data well, underpredicting cell growth in the early stage while overpredicting in the later stage, as shown in Figure 4 (solid line). More importantly, DFBA Lab cannot predict the unknown interspecies interactions within the coculture due to the lack of a shared community compartment. One can only manually add the known cross-feeding mechanisms, such as CO_2_ and O_2_ cross-feeding, in the M-P coculture.

**Table 4:** Growth- and non-growth-associated maintenance energy (GAM and NGAM) parameters used in the DFBA Lab models

	**Cyanobacteria**		**Methanotroph**	
	**GAM**	**NGAM**	**GAM**	**NGAM**
**Single culture**	60	0.6	23	10.6
**Coculture**	40	0.05	23	5.6

GAM: Growth associated maintenance energy; NGAM: non-growth associated maintenance energy.

### DynamiCom

The experimental data reported previously (figure 4 of our previous work^[22]^) for the gas composition of 60% CH_4_, 30% CO_2_ and 10% N_2_ were used for DynamiCom simulation. In DynamiCom, the semi-structured kinetic model completely determines the system dynamics, and there is no feedback from the GEM to the kinetic model. Therefore, guaranteed by the accuracy of the semi-structured kinetic model^[22]^, the coculture growth predicted by DynamiCom over time showed excellent agreement with experimental data. For each time instant, the predictions from the semi-structured kinetic model (i.e., the substrate uptake rates and product excretion rates for each organism in the coculture) serve as the additional constraints to regulate the predictions from SteadyCom; then the interspecies interactions predicted by SteadyCom are recorded to track the dynamic evolution of the interspecies interactions within the coculture. Because the communication between the kinetic model and SteadyCom is unidirectional, the constraints generated by the kinetic model can be applied at any desired time interval or frequency. To reduce computation, one can specify the output interval for the numerical solver employed to solve the ordinary differential equations (ODEs) in the kinetic model.

**Figure 5:**
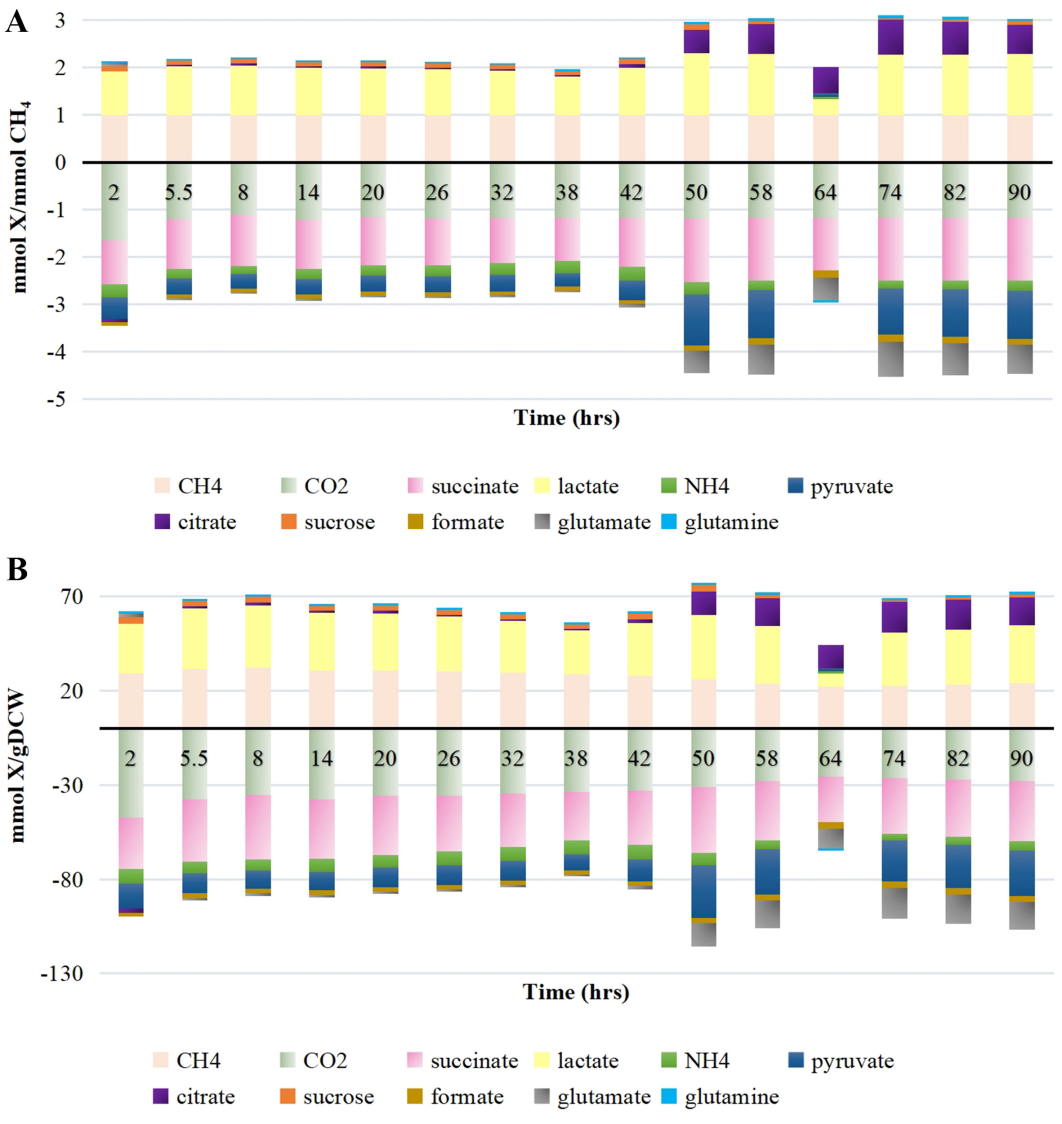
Fluxes of the top exchanged metabolite over time normalized by the (A) Ch4 uptake rate, or (B) total coculture growth rate.

Similar to SteadyCom, the top exchanged metabolites predicted by DynamiCom under different *in silico* setups (i.e., what key central carbon metabolites were allowed to be exchanged) were consistent. In addition, driven by the evolving constraints from the kinetic model throughout the batch growth, the interspecies interactions predicted by SteadyCom also evolve continuously. The top exchanged metabolites over time are plotted in Figure 5 to demonstrate their dynamic nature throughout the batch growth. CH_4_ and CO_2_ consumption rates are included to depict the comparison between the main carbon sources and the exchanged metabolites. Figure 5A shows the fluxes normalized by CH_4_ uptake rate (mmol X/mmol CH_4_ where X denotes a metabolite); Figure 5B shows the fluxes normalized by total coculture growth rate (mmol X/gDCW). In these figures, the positive flux of a metabolite indicates that the metabolite was produced by methanotrophs, while the negative flux indicates that the metabolite was produced by cyanobacteria.

Figure 5 suggests that approximately after 38 h (establishing the mutualistic relationship), cyanobacteria produced more pyruvate and glutamate and kept providing NH_4_, succinate, and formate for methanotrophs. On the other hand, methanotrophs produced more citrate and slightly more glutamine and kept providing lactate and sucrose for cyanobacteria.

**Figure 6:**
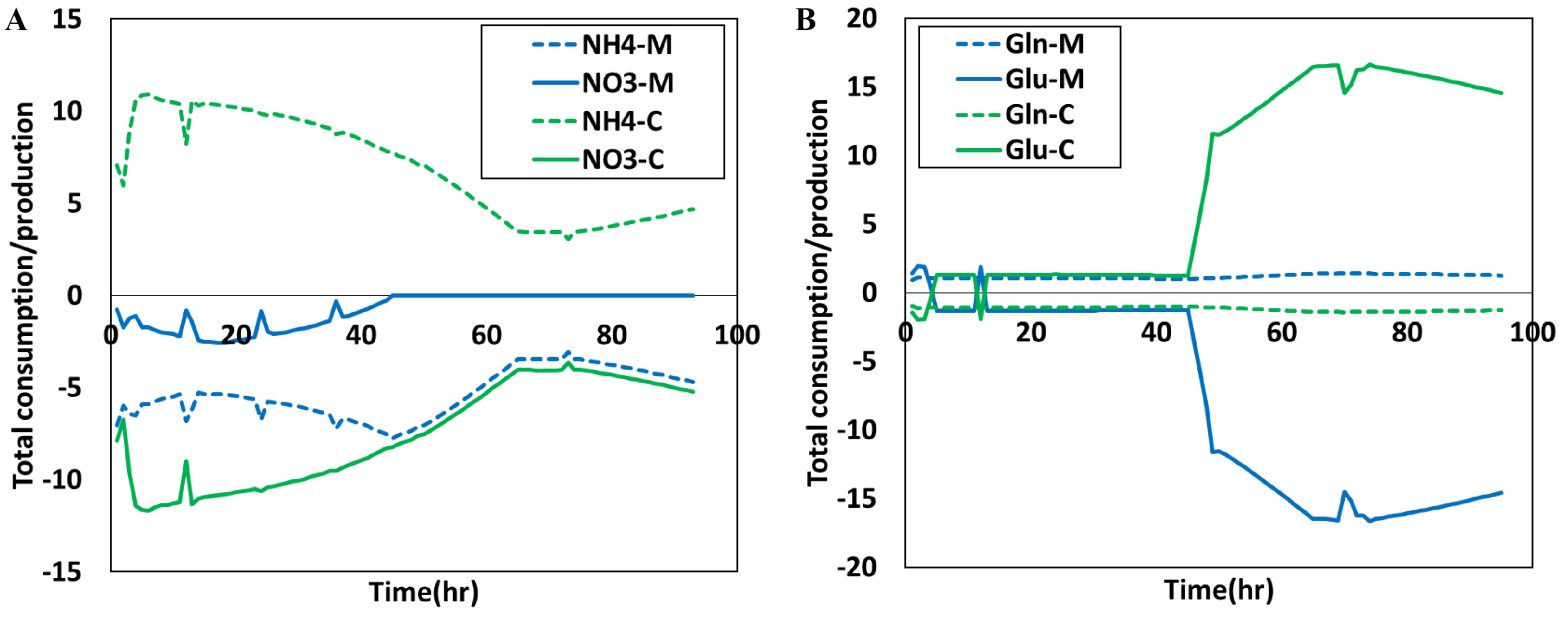
(A) Methanotrophs initially consume both nitrate (from the culture medium) and ammonium (produced by cyanobacteria), but consume only ammonium produced by *A. platensis* after ~45 h. This dynamic evolution coincides with the significant increase in glutamate exchange (Glu-M and Glu-C) within coculture, as shown in (B). Positive values indicate production while negative values indicate consumption. The irregularities (i.e., the non-smooth parts) in the prediction are the consequence of the change of CH_4_/CO_2_ uptake by the species during and after refeeding the system.

The exchange fluxes of ammonium, nitrate, glutamate, and glutamine over time are plotted in Figure 6, which clearly illustrates the dynamic evolution of the emergent metabolic interactions within the coculture of *M. buryatense* and *A. platensis*. Figure 6 suggests that right after inoculation, the methanotroph in the coculture consumes both nitrate (from the culture medium) and ammonium (produced by the cyanobacteria). However, after about 45 h, *M. buryatense* exclusively consumes ammonium produced by *A. platensis*, which coincides with the significant increase in glutamate exchange within coculture.

Again, it is important to note that the base unit for flux in the coculture GEM is the unit mass of the coculture, not any individual species. Even though the flux of ammonium production by *A. platensis* is equal to the flux of ammonium consumption by *M. buryatense*, the flux for each individual species is quite different, as the coculture consists of about 80% of cyanobacteria and 20% of methanotrophs.

## DISCUSSION AND CONCLUSION

As shown above, DynamiCom not only consistently predicted the metabolic interactions within the M-P coculture, but also predicted the establishment of the emergent mutualistic N-exchange between the methanotroph and cyanobacteria. While we are in the process of conducting more experimental validations, we would like to note that there is ample evidence in the previously published experimental work that supports key aspects of the predictions from both SteadyCom and DynamiCom. In general, in photoautotroph-bacteria cocultures, photoautotrophs provide O_2_ and organics through photosynthesis for bacterial consumption, whereas the bacteria produce CO_2_ and inorganic substances through respiration to sustain photoautotroph growth^[66]^. It is now recognized that bacteria secrete micronutrient metabolites such as vitamin B_12_, phytohormones (IAA, abscisic acid, cytokinins, ethylene, and gibberellins), thiamine derivatives, and siderophores to accelerate photoautotroph metabolism and biomass growth^[67,68]^. However, the metabolite exchanges within photoautotroph-bacteria coculture are not limited to micronutrients. Macronutrients such as nitrogen-mediated interactions also occur between photoautotrophs and bacteria. Recently, de-Bashan *et al.* clearly showed that co-evolution is not a prerequisite for a functioning synthetic mutualism between a microalga and a bacteria^[69]^. Using highly specific analytical tools capable of analyzing single cells within the association, such as NanoSIMS isotopic imaging and fluorescent in situ hybridization (FISH), combined with enforcing initial proximity between cells of the two species in alginate beads, they directly showed that C and N containing compounds were exchanged during interaction and association, which is beneficial to both microorganisms as demonstrated by their mutually enhanced growth. Furthermore, it is important to note that this association was man-made and created almost spontaneously without long-term co-evolution, which is a similar case to the coculture studied in this work.

It has been shown that cyanobacteria can produce formate through the action of pyruvate formate lyase without the associated production of NADH or reduced ferredoxin^[70]^. In addition, Riccardi *et al.* showed that the biosynthetic pathways in cyanobacteria are highly responsive to specific exogenous amino acids, suggesting it is possible that the cyanobacteria in the coculture would be able to recognize the presence of amino acid in bulk (produced by methanotrophs) and would downregulate its own amino acids production^[71]^. On the other hand, Zhu *et al.* showed that methanotrophs could excrete citrate^[72]^, while Gilman *et al.* showed that *M. buryatense* 5GB1 can produce lactate and succinate^[59]^. In addition, it was shown that *Methylomicrobium alcaliphilum* 20Z, a closely related methanotroph strain to *M. buryatense* 5GB1, can produce sucrose. Finally, the predicted emergent N-exchange has been observed in a cyanobacteria-heterotroph biofilm using nanoscale secondary ion mass spectrometry (NanoSIMS) image analysis^[73]^. By doping the cyanobacteria consortia biofilm with^15^*N*-labeled nitrogen source (^15^NH_4_^+^ or ^15^NO_3_^-^), it was observed that the heterotrophs in the cyanobacterial consortium biofilm only uptake NH_4_^+^, but not NO_3_^-^. Another recent study investigated a stable mutualism between *C. sorokiniana* and *Saccharomyces cerevisiae*, isolated from winery wastewater, under synthetic growth conditions^[74]^. They observed a mutualistic relationship based on carbon (C) and nitrogen (N) cross-feeding, where microalgae consume CO_2_ produced by the yeast, while providing ammonium (derived from inaccessible nitrite) to the yeast as its N source.

As discussed in the Introduction section, elucidating interspecies interactions within microbiomes is very challenging due to the complexity of the system, as well as the lack of tractability of the microbiome. For example, if an exchanged metabolite is rate-limiting, such as O_2_ in the M-P coculture, one may not detect the metabolite in the coculture at all as the metabolite produced by one partner would be immediately and completely consumed by the other. For these reasons, the inter- and intracellular details predicted by the GEM for the microbiome can help address these challenges effectively. For the case of the M-P coculture, the predictions provided by DynamiCom offer valuable insights for generating hypotheses on the fundamental principles that drive interspecies interactions. The predictions also provide guidance on designing experiments to validate these hypotheses. It is important to note that in DynamiCom, the inter-species interactions predicted by SteadyCom are constrained by the cross-membrane fluxes predicted by the semi-structured kinetic model. In other words, the accuracy of the kinetic model provides the foundation for the relevance of the DynamiCom prediction. Therefore, it is important to validate the accuracy of the kinetic model before using its prediction to regulate the inter-species predictions.

As the first attempt to understand the dynamic evolution of inter-species interactions within the M-P coculture, it is worth noting that there are also some limitations with DynamiCom. One limitation is the use of a modified SteadyCom to predict the metabolic details of interspecies interactions. SteadyCom assumes the coculture has reached a steady state and all members of the community grow at the same rate (i.e., the community composition does not change over time). However, this is usually not the case for synthetic coculture, which may skew the predicted establishment of the interactions, especially during the initial phase of coculture growth. The other limitation is the scope of GEM for each strain in the coculture. Currently, the GEMs used for the model M-P coculture only contain primary metabolites. However, many reported cross-feeding metabolites are secondary metabolites, which were not included in the model and therefore cannot be predicted. These limitations will be addressed in our future research.

In conclusion, we developed a new dynamic GEM approach, DynamiCom, for a model coculture, *M. buryatense* 5GB1 - *A. platensis*, which is capable of predicting the emergent inter-species metabolic interactions. As available GEMs, especially the ones for non-model organisms, only contain stoichiometric information of the cellular metabolic network without any regulatory mechanisms or information, adding validated constraints could improve the accuracy of the model prediction. In this work, we used the predictions from a validated kinetic model of the coculture, i.e., individual substrate consumption rates and product excretion rates, as additional constraints to regulate the prediction of SteadyCom. It enabled DynamiCom to consistently predict the top metabolites being exchanged and the establishment of the emergent mutualistic N-P exchange within the coculture. The model predictions are supported by a plethora of literature reports on methanotrophs, cyanobacteria, and cyanobacteria-heterotroph cocultures.

## DECLARATIONS

### Availability of data and materials

Experimental data used in the work can be found at: https://github.com/AU-Wang-He-Group/Semi-structured-KineticModel. The codes for all the modeling work can be found at: https://github.com/AU-Wang-He-Group/DynamicGEM-coculture.

### Copyright

© The Author(s) 2024.
