# Supplementary material for: Probing interspecies metabolic interactions within a synthetic binary microbiome using genome-scale modeling
Source: Microbiome Res Rep. 2024 May 27;3(3):31. doi: 10.20517/mrr.2023.70 (PMC11480724; doi:10.20517/mrr.2023.70)
Supplement: Supplementary file 1 [file mrr-3-3-31-SupplementaryMaterials.pdf]

Supplementary Materials

Probing interspecies metabolic interactions within a synthetic binary microbiome using genome-scale modeling

Kiumars Badr, Q. Peter He, Jin Wang

Department of Chemical Engineering, Auburn University, Auburn, AL 36849, USA.

Correspondence to: Prof. Jin Wang, Department of Chemical Engineering, Auburn University, 222 Foy Union Cir, Auburn, AL 36849, USA. E-mail: wang@auburn.edu

Supplementary Table 1. Major modifications made to GEM NIES-39

Reaction	Modification	Description and reason
6 h + 2 fdrd + pq -> 2 fdox + 4 h[t] + pqh2	added	Photosynthesis and electron transport chain (Peltier, Aro, and Shikanai 2016)
atp + f6p -> adp + fdp + h	blocked	Calvin cycle / Pentose phosphate pathway; inactivated by light (Plaxton 1996)
g6p + nadp -> 6pgl + h + nadph	blocked	Pentose phosphate pathway; light inhibits it (Plaxton 1996)
fdp + h2o -> f6p + pi	blocked	Calvin cycle / Pentose phosphate pathway; light inactivates it (Lemaire 2004)
ser-L -> nh4 + pyr	blocked	Serine metabolism; This reaction is not present (KEGG)
glu-L + h2o + nadp <=> akg + h + nadph + nh4	Upper bound=0	Glutamate metabolism; This reaction is irreversible reaction (Muro-Pastor 2005)
gtp + h2o -> gmp + h + ppi	blocked	This reaction is not present (KEGG) Pyrimidine metabolism
dgtp + h2o -> dgmp + h + ppi	blocked	This reaction is not present (KEGG)
dutp + h2o -> dump + h + ppi	blocked	This reaction is not present (KEGG)
h2o + utp -> h + ppi + ump	blocked	This reaction is not present (KEGG)
10fthf + h2o -> for + h + thf	blocked	This reaction is not present (KEGG) One carbon pool by folate

**Metabolites**	**Name**
f6p	D-fructose-6-phosphate
fad	Flavin adenine dinucleotide oxidized
g6p	D-glucose-6-phosphate
6pgl	6-phospho-D-glucono-1-5-lactone
ser-L	L-Serine
pyr	Pyruvate
glu-L	L-Glutamate
akg	2-Oxoglutarate
10fthf	10-Formyltetrahydrofolate
thf	Tetrahydrofolate
for	Formate

Bibliography

Peltier, Gilles, Eva-Mari Aro, and Toshiharu Shikanai. 2016. “NDH-1 and NDH-2 Plastoquinone Reductases in Oxygenic Photosynthesis.” *Annual Review of Plant Biology* 67:55–80.

Plaxton, William. 1996. “The Organization and Regulation of Plant Glycolysis.” Annual review of plant physiology and plant molecular biology. 47. 185-214.

Lemaire C, Guibet-Grandmougin F, Angles D, Dujardin G, Bonnefoy N. 2004. “A yeast mitochondrial membrane methyltransferase-like protein can compensate for oxa1 mutations.” *J Biol Chem*, 279:47464-47472.

Muro-Pastor MI, Reyes JC, Florencio FJ. 2005. “Ammonium assimilation in cyanobacteria.” *Photosynth Res*. 83(2):135-150.
